# Supplementary material for: 18F-Facbc in Prostate Cancer: A Systematic Review and Meta-Analysis
Source: Cancers (Basel). 2019 Sep 11;11(9):1348. doi: 10.3390/cancers11091348 (PMC6769578; doi:10.3390/cancers11091348)
Supplement: Supplementary file 1 [file cancers-11-01348-s001.pdf]

Supplementary Materials: ^{18}F-Facbc in Prostate Cancer: A Systematic Review and Meta-Analysis

Riccardo Laudicella, Domenico Albano, Pierpaolo Alongi, Giovanni Argiroffi, Matteo Bauckneht, Sergio Baldari, Francesco Bertagna, Michele Boero, Giuseppe De Vincentis, Angelo Del Sole, Giuseppe Rubini, Lorenzo Fantechi, Viviana Frantellizzi, Gloria Ganduscio, Priscilla Guglielmo, Anna Giulia Nappi and Laura Evangelista, on the behalf of Young AIMN Working Group

Table 1. QUADAS 2 score for each selected study (green smile = low risk; red smile = high risk; yellow question mark = unclear).

N	Author, Year (ref)	Risk of Bias				Applicability Concerns		
		**Patient Selection**	**Index Test**	**Reference Standard**	**Flow and Timing**	**Patient Selection**	**Index Test**	**Reference Standard**
1	Schuster et al. 2007 [20]	:)	:)	:(	:)	:)	:)	:)
2	Schuster et al. 2011 [21]	:)	:)	:)	:)	:)	:)	:)
3	Turkbey et al. 2014 [22]	:)	?	:)	:)	:)	:)	:)
4	Kairemo et al. 2014 [23]	:)	:(	:(	:(	:)	:)	:)
5	Nanni et al. 2014 [24]	:)	:(	:)	:)	:)	:)	:)
6	Nanni et al. 2015 [25]	:)	:)	:(	:(	:)	:)	:)
7	Odewole et al. 2016 [26]	:)	:)	:)	:)	:)	:)	:)
8	Bach-Gansmo et al. 2017 [15]	?	:)	:(	:)	:)	:)	:)
9	Akin-Akintayo et al. 2017 [27]	:)	:)	:(	:(	:)	:)	?
10	Selnaes et al. 2018 [28]	:)	:)	:)	:)	:)	:)	:)
11	Jambor et al. 2018 [29]	:)	:)	:)	:)	:)	:)	:)
12	Akin-Akintayo et al. 2018 [30]	:)	:)	:)	:)	:)	:)	:)
13	Andriole et al. 2019 [31]	:)	?	:(	:(	:)	:)	:)
14	England et al. 2019 [32]	:)	?	:(	:(	:)	:)	:)
15	Suzuki et al. 2019 [33]	:)	:)	:)	:)	:)	:)	:)

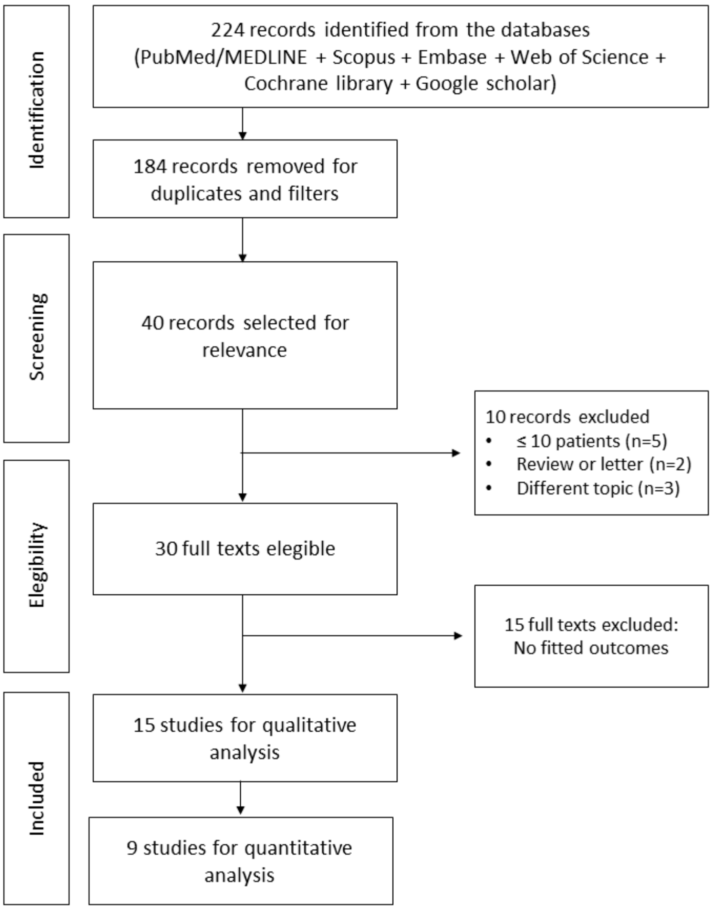

Figure S1. Forest-plots for the patient-based and region-based analysis.

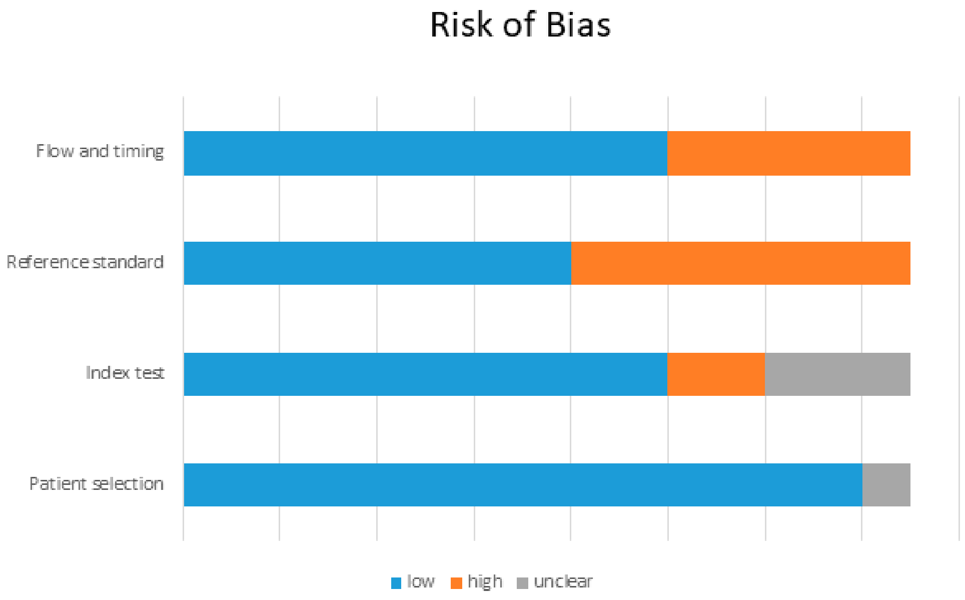

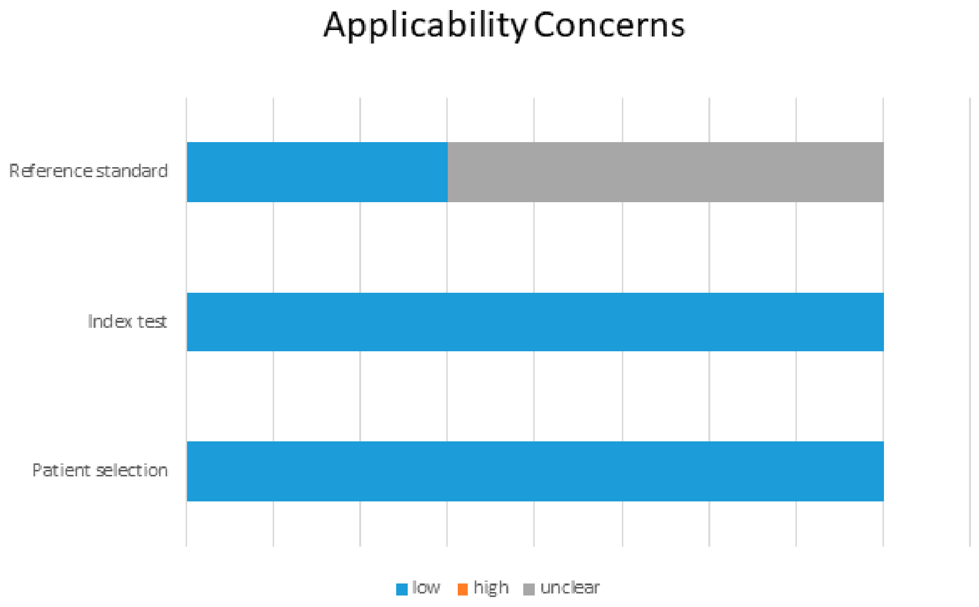

Figure S2. ROC curves for patient-based and region-based analyses.

© 2019 by the authors. Licensee MDPI, Basel, Switzerland. This article is an open access article distributed under the terms and conditions of the Creative Commons Attribution (CC BY) license (http://creativecommons.org/licenses/by/4.0/).
